# Fibroblast Culture Cells Keep Track of Circadian Rhythms

**DOI:** 10.1371/journal.pbio.0030348

**Published:** 2005-09-27

Embedded in the brain's hypothalamus on top of the intersection of the two optic nerves lies a group of some 10,000 cells known as the suprachiasmatic nucleus. This neural region determines the biological equivalent of Greenwich Mean Time: the circadian cycle. Circadian rhythms influence phenomena as diverse as leaf position in plants, fatigue patterns in mice, and late-night hunger in human adolescents. By measuring exactly how long an organism takes to complete one cycle, scientists hope to gain insight into the mechanisms underlying these broad effects. “Circadian” translates from Latin to “approximately a day,” but scientists search for more precise answers about how genes and environment account for fluctuations in circadian cycle length.

Theoretically, scientists could measure the circadian signal from its source in the suprachiasmatic nucleus—but no method exists to do this in a living subject. Instead, scientists rely on prolonged observation of live mice or humans. They document when mice use their mouse wheel, for example, and when they sleep. But to screen for and identify circadian rhythm variations in humans, the required period of lengthy observation is prohibitively costly and labor intensive.[Fig pbio-0030348-g001]

**Figure pbio-0030348-g001:**
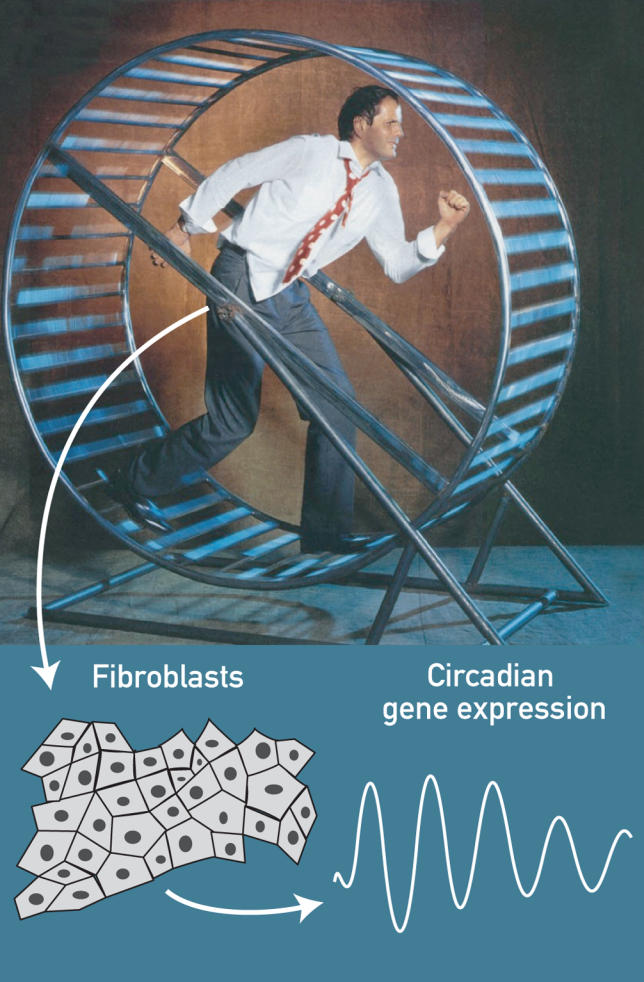
Studying circadian rhythms in humans isn't as straightforward as it is with lab rodents. As a proxy for humans, researchers compared circadian gene expression in primary fi broblasts from different human individuals and found unexpectedly large differences between the circadian clocks of different human subjects

Circumventing these technical limitations, Ueli Schibler and colleagues have recently designed a method to measure circadian cycles in mammalian cells cultured from tissues other than the suprachiasmatic nucleus. The researchers infected human and mouse fibroblast tissue cultures with a virus engineered to report when a certain host circadian rhythm gene was expressed. They found that their data jibed with the previously accepted length of the human circadian cycle: 24.5 hours. Because of the sensitivity of their method, Schibler and colleagues also confirmed that, for both humans and mice, circadian rhythms vary substantially between individuals. This suggests that the genetics of the circadian clock likewise varies between individuals.

The so-called reporter gene, lucerifase, produces a protein that illuminates the cell. Steve Brown, a postdoc in Schibler's laboratory, created a virus system that specifically inserts lucerifase into the host genome near a gene important for establishing the circadian rhythm. With this system, the same gene that promotes expression of the circadian rhythm gene also promotes expression of lucerifase—consequently, the fibroblast cultures light up in time with the circadian clock.

Schibler and colleagues obtained human fibroblast skin cells from the abdomen, buttocks, male foreskin, and other areas to measure the circadian rhythm indirectly. Unlike an individual's true rhythm, a fibroblast cell culture's rhythm does not vary with changes in light exposure or sleep habits. The researchers point out that their method can expose differences in circadian rhythms; it does not, however, directly measure the signal from the suprachiasmatic nucleus that coordinates each individual cell's clock.

The authors also found that circadian rhythm variations between cultures from the same individuals differed far less than differences between individuals. This discovery indicates that fibroblast cell cultures are reliable tools for approximating an individual's genetically determined circadian rhythm. The scientists also found that, for mice, circadian rhythm time obtained from prolonged observation and that obtained from the new viral method were showing the same tendency, although the differences observed in behavior were exacerbated in molecular fibroblast rhythms. This result suggests that the fibroblast circadian clocks might give relevant information about the brain's circadian clock in humans as well as in mice.

In the future, scientists may use the new method to screen large populations for genetically linked sleep disturbances such as advanced and delayed sleep phase syndromes. They may also use this test to home in on the genetic mechanism responsible for such conditions. Outside the realm of medicine, future genetic studies of circadian rhythm may exploit the method developed by Schibler and colleagues to explore questions about the exact relationship between the suprachiasmatic nucleus and the circadian rhythms of cultured fibroblast cells. Just how does this brain structure coordinate genetic imperatives with environmental input including light fluctuations? Finally, frequent fliers, bleary-eyed in foreign time zones, may get an answer to why waking up is so hard to do. —*Jessica Tanenbaum*

